# Disease Burden of *Clostridium difficile* Infections in Adults, Hong Kong, China, 2006–2014

**DOI:** 10.3201/eid2310.170797

**Published:** 2017-10

**Authors:** Jeffery Ho, Rudin Z.W. Dai, Thomas N.Y. Kwong, Xiansong Wang, Lin Zhang, Margaret Ip, Raphael Chan, Peter M.K. Hawkey, Kelvin L.Y. Lam, Martin C.S. Wong, Gary Tse, Matthew T.V. Chan, Francis K.L. Chan, Jun Yu, Siew C. Ng, Nelson Lee, Justin C.Y. Wu, Joseph J.Y. Sung, William K.K. Wu, Sunny H. Wong

**Affiliations:** Chinese University of Hong Kong, Hong Kong, China (J. Ho, R.Z.W. Dai, T.N.Y. Kwong, X. Wang, L. Zhang, M. Ip, R. Chan, K.L.Y. Lam, M.C.S. Wong, G. Tse, M.T.V. Chan, F.K.L. Chan, J. Yu, S.C. Ng, N. Lee, J.C.Y. Wu, J.J.Y. Sung, W.K.K. Wu, S.H. Wong);; Health Protection Agency, Birmingham, UK (P.M.K. Hawkey);; University of Birmingham, Birmingham (P.M.K. Hawkey)

**Keywords:** Clostridium difficile, bacteria, infections, pseudomembranous colitis, surveillance, epidemiology, disease burden, enteric infections, Hong Kong, China

## Abstract

Cross-sectional studies suggest an increasing trend in incidence and relatively low recurrence rates of *Clostridium difficile* infections in Asia than in Europe and North America. The temporal trend of *C. difficile* infection in Asia is not completely understood. We conducted a territory-wide population-based observational study to investigate the burden and clinical outcomes in Hong Kong, China, over a 9-year period. A total of 15,753 cases were identified, including 14,402 (91.4%) healthcare-associated cases and 817 (5.1%) community-associated cases. After adjustment for diagnostic test, we found that incidence increased from 15.41 cases/100,000 persons in 2006 to 36.31 cases/100,000 persons in 2014, an annual increase of 26%. This increase was associated with elderly patients, for whom incidence increased 3-fold over the period. Recurrence at 60 days increased from 5.7% in 2006 to 9.1% in 2014 (p<0.001). Our data suggest the need for further surveillance, especially in Asia, which contains ≈60% of the world’s population.

*Clostridium difficile* infection (CDI) is a major cause of nosocomial illness worldwide. CDI is associated with high rates of mortality and illness (*1*) and has a case-fatality rate of up to 14% within 30 days after diagnosis (*2*). Disease recurrence further increases illness rates, reduces quality of life, and might occur in up to 27% of the incident cases (*3*). These features place a major burden on healthcare systems.

The disease burden of CDI has been well studied in Europe and North America. Although antimicrobial drug stewardship programs have contributed to a decrease in incidence in some countries, such as the United Kingdom and Finland (*2*,*4*), CDI is still a major health burden in other countries. In South Korea, a nationwide study showed an increasing trend in incidence of CDI (*5*). Disease occurrence in the United States has doubled during 2001–2010 (*6*). A similar trend of increase was also observed in a prospective surveillance study in Australia (*7*) and a retrospective observational study in Germany (*8*).

Emergence of community-associated CDI (CA-CDI), which originates in a community without traditional risk factors (*9*), is also a concern. A major proportion of CDI cases was attributable to a community in the United States (*1*). In Finland, episodes of CA-CDI have shown a major increase versus an overall decrease in disease incidence (*2*). Surveillance of CDI cases diagnosed during admission of patients showed that up to 50% of CDI cases were community associated (*3*,*7*).

Although Asia contains ≈60% of the world’s population, epidemiologic data of CDI for this continent are sparse (*10*). The disease burden and secular trend of incidence, especially CA-CDI, has not been reported for this region. Therefore, we conducted a large territory-wide study to investigate the disease burden and clinical outcomes of CDI in Hong Kong, China.

## Methods

### Study Population and Case Identification

We identified all patients in Hong Kong given a diagnosis of CDI during January 1, 2006–December 31, 2014, from the Clinical Data Analysis and Reporting System (CDARS), which is a computerized database of patient records managed by the Hong Kong Hospital Authority. The database contains laboratory and clinical information, including patient demographics, disease diagnoses, investigations, procedures, and drug prescription records in the public hospital system. It also contains information regarding residence in homes for elderly persons and medical care provided by the Community Geriatric Assessment Team in the ambulatory care setting. This public hospital system is composed of 41 hospitals within 7 service clusters and provides >90% of inpatient medical services in the territory. There were >1 million inpatient discharges and deaths in this system during 2013–2014. In addition to the ward-based services, the system also provides the outpatient clinics and geriatric ambulatory care in Hong Kong. This electronic database has been used for conducting robust population studies (*11*,*12*).

We defined a case of CDI as a positive result on culture, toxin, or molecular assay for a diarrheal stool specimen obtained from an inpatient resident >18 years of age. Patients with samples obtained >48 hours after admission or those who were hospitalized in a healthcare facility within the previous 4 weeks were classified as having cases of healthcare-associated CDI (HA-CDI). Patients who had not been hospitalized in a healthcare facility within the previous 12 weeks were classified as having cases of CA-CDI. Patients who had been hospitalized in a healthcare facility (including long-term care facilities, such as homes for elderly persons and palliative care centers) within the previous 4–12 weeks were classified as indeterminate. We defined an incident case as a CDI episode without a positive laboratory test result in the previous 60 days.

### Data Extraction

We obtained anonymized clinical information, including patient demographics, disease diagnoses, laboratory results, and clinical outcomes. Patient demographic data included age, sex, and residence in homes for elderly persons. Relevant disease diagnoses were identified by using codes from the International Classification of Diseases, 9th Revision (https://www.cdc.gov/nchs/icd/icd9.htm), including inflammatory bowel disease (555._–556._), Crohn’s disease (555._), ulcerative colitis (556._), colectomy (45.7 or 45.71–45.79), or surgical intervention during the same admission, and other concurrent conditions.

We retrieved data on medication prescriptions, including antimicrobial drug use within 8 weeks before CDI diagnosis. Medications prescribed under hospital authority–affiliated clinics and long-term care facilities were accessible in the database. Severe CDI was defined by either a maximum leukocyte count >15,000 cells/μL or a >50% increase in serum creatinine level, according to Cohen et al. (*13*). These data were captured from CDARS from 1 day before to 7 days after the CDI diagnosis date. Refractory disease referred to nonresponding disease requiring >14 days of continued treatment. We defined death as dying within 30 days after the diagnosis of CDI and recurrence as a recurrent diarrheal stool specimen with a positive test result for *C. difficile* within 60 days after completion of CDI treatment.

### Statistical Analysis

We reported descriptive statistics as median, interquartile range (IQR), and percentage and calculated the overall crude incidence of CDI as the number of patients given a diagnosis of CDI/100,000 persons >18 years of age. The midyear population was obtained from Hong Kong Census and Statistics Department.

We also estimated incidences of health-associated and community-associated cases in the same manner. We used the χ^2^ test for trend to compare differences in incidences, mortality rates, and recurrence rates. Assuming a Poisson distribution, we calculated 95% CIs for the incidence rate. We analyzed potential predictors for 30-day mortality rate and 60-day recurrence rate by using univariate and multivariate forward Wald logistic regression. We used Cox proportional hazard regression to identify factors that decreased the time to recurrence after an episode. We also used SPSS for Windows version 22.0 (IBM Corp., Armonk, NY, USA) to perform statistical analysis.

### Ethical Statement

This study was conducted in accordance with the Declaration of Helsinki (2013 version) and approved by the Joint Clinical Research Ethics Committee of the Chinese University of Hong Kong and Hospital Authority New Territory East Cluster. All clinical data were anonymized by the CDARS, and all potential patient identifiers were removed upon return of database searches.

## Results

### Disease Burden, Incidence, and Clinical Outcomes

**Figure 1:**
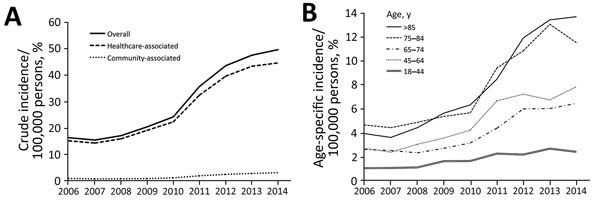
*Clostridium difficile* infections in adults, Hong Kong, China, 2006–2014. A) Crude incidence of healthcare-associated and community-associated *C. difficile* infections increased significantly (p<0.001 by χ^2^ test for trend). B) Incidence of infections, by age group.

We identified 15,753 CDIs during 2006−2014. These infections included 14,402 (91.4%) healthcare-associated and 817 (5.1%) community-associated infections. The remaining 534 infections were indeterminate. The median age of case-patients was 78 (range 64–86) years, and there were more women (51.6%) in the entire cohort. The diagnostic test-adjusted incidence increased significantly from 15.41 cases/100,000 persons in 2006 to 36.31 cases/100,000 persons in 2014 (p<0.01 by χ^2 ^test for trend). We observed the trend of increase across all age groups. However, the incidence for elderly persons (>65 years of age) increased by the largest margin from 12.98 cases/100,000 persons in 2006 to 35.11 cases/100,000 persons in 2014 (Figure 1). The incidence of CA-CDI increased by more than 4-fold from 0.86 cases/100,000 persons in 2006 to 2.96 cases/100,000 persons in 2014. Incidence of HA-CDI increased annually by an average of 26%, and incidence of CA-CDI increased annually by an average of 29% (Table 1). 

**Table 1:** Estimated crude incidence of *Clostridium difficile* infections, Hong Kong, China, by epidemiologic category, 2006–2014*

Year	Adult population†	No. (%) CDI‡			Incidence/100,000 persons					
					Crude			Adjusted§		
		Overall	HA-CDI	CA-CDI	Overall	HA-CDI	CA-CDI	Overall	HA-CDI	CA-CDI
2006	5,571,096	903	836 (92.58)	48 (5.32)	16.21	15.01	0.86	15.41	14.26	0.82
2007	5,553,789	849	786 (92.58)	35 (4.12)	15.29	14.15	0.63	14.35	13.29	0.59
2008	5,635,881	953	888 (93.18)	41 (4.30)	16.91	15.76	0.73	15.53	14.68	0.67
2009	5,711,689	1,156	1,082 (93.60)	42 (3.63)	20.24	18.94	0.74	23.11	21.63	0.84
2010	5,788,704	1,389	1,284 (92.44)	62 (4.46)	24.00	22.18	1.07	20.77	19.19	0.93
2011	5,865,870	2,079	1,890 (90.91)	105 (5.05)	35.44	32.22	1.79	23.40	21.28	1.18
2012	5,943,512	2,576	2,341 (90.88)	141 (5.47)	43.34	39.39	2.37	33.32	30.28	1.82
2013	6,014,771	2,844	2,594 (91.21)	163 (5.73)	47.28	43.13	2.71	34.71	31.66	1.99
2014	6,085,892	3,004	2,701 (89.91)	180 (5.99)	49.36	44.38	2.96	36.31	39.90	2.18
Overall¶	NA	NA	NA	NA	29.46 (15.85–43.07)#	26.94 (14.79–39.09)	1.51 (0.63–2.39)	24.10 (17.42–30.77)	22.91 (15.86–29.96)	1.22 (0.76–1.69)

*CDI, *Clostridium difficile* infection; CA-CDI, community-associated CDI; HA-CDI, healthcare-associated CDI; NA, not applicable. †Midyear population >18 years of age reported by the Census and Statistics Department of Hong Kong. ‡There were 534 indeterminate cases according to criteria of Cohen et al (*13*): n = 19 (2006); n = 28 (2007); n = 24 (2008); n = 32 (2009); n = 43 (2010); n = 84 (2011); n = 94 (2012); n = 87 (2013); n = 123 (2014). §Overall incidence adjusted for diagnostic method use, assuming equal sensitivity across tests. The 2010 cohort was used as the reference population. ¶Values in parentheses in this row are 95% CIs. #p<0.01 by χ^2^ test for trend.

The overall 30-day mortality rate was 22.5%. The disease recurrence rate was 7.8% at 60 days after completion of initial CDI treatment. A total of 30.2% of the patients lived in a home for the elderly, and most (79.4%) patients had visited a healthcare facility <4 weeks before disease onset. Most (>75.6%) patients had taken an antimicrobial drug known to be associated with medium to high risk for CDI before disease onset (*14*,*15*). A total of 47.8% had taken a proton-pump inhibitor, and 47.3% had taken a histamine-2 antagonist (Table 2).

**Table 2:** Characteristics of patients with *Clostridium difficile* infections, Hong Kong, China, 2006–2014*

Characteristic	No. (%) patients		
	Overall, n = 15,753†	HA-CDI, n = 14,402	CA-CDI, n = 817
Age, y			
<44	1,040 (6.6)	893 (6.2)	105 (12.9)
45–64	2,930 (18.6)	2,621 (18.2)	203 (24.8)
65–84	7,026 (44.6)	6,495 (45.1)	301 (36.9)
>85	4,757 (30.2)	4,393 (30.5)	208 (25.4)
Sex			
M	7,624 (48.4)	7,028 (48.8)	337 (41.2)
F	8,129 (51.6)	7,374 (51.2)	480 (58.8)
Resident of home for elderly persons	4,757 (30.2)	4,393 (30.5)	203 (24.9)
Severe disease	6,868 (43.6)	6,294 (43.7)	340 (41.6)
Antimicrobial drug use‡			
High-risk drug	10,397 (66.0)	9,822 (68.2)	299 (36.6)
Medium-risk drug	11,909 (75.6)	11,320 (78.6)	318 (38.9)
Low-risk drug	221 (1.4)	216 (1.5)	74 (1.4)
Diagnostic test			
Bacterial culture	4,883 (31.0)	4,421 (30.7)	259 (31.7)
Toxin detection	5,246 (33.3)	4,940 (34.3)	195 (23.9)
NAAT	5,624 (35.7)	5,041 (35.0)	363 (44.4)
Use of proton-pump inhibitor	7,530 (47.8)	7,086 (49.2)	180 (22.0)
Use of histamine-2 receptor antagonist	7,451 (47.3)	6,927 (48.1)	273 (33.4)
Concurrent condition			
Myocardial infarction	1,497 (9.5)	1,411 (9.8)	29 (3.6)
Cerebrovascular disease	5,183 (32.9)	4,825 (33.5)	176 (21.6)
Chronic lung disease	2,410 (15.3)	2,232 (15.5)	100 (12.2)
Diabetes mellitus	2,899 (18.4)	2,693 (18.7)	103 (12.6)
Renal disease	3,592 (22.8)	3,341 (23.2)	113 (13.8)
Nonmetastatic tumor	3,970 (25.2)	3,701 (25.7)	134 (16.4)
AIDS	79 (0.5)	58 (0.4)	13 (1.6)
Inflammatory bowel disease	95 (0.6)	58 (0.4)	36 (4.4)
Deaths			
During hospital stay	3,733 (23.7)	3,528 (24.5)	51 (6.2)
30-d all-cause	3,544 (22.5)	3,341 (23.2)	80 (9.8)
60-d all-cause	5,088 (32.3)	4,781 (33.2)	106 (13.0)
Recurrence, d§			
30	961 (6.1)	907 (6.3)	17 (2.1)
60	1,229 (7.8)	1,152 (8.0)	25 (3.0)
90	1,339(8.5)	1,267 (8.8)	28 (3.4)
180	1,481(9.4)	1,397 (9.7)	33 (4.0)

*CA-CDI, community-associated *C. difficile* infection; CDI; HA-CDI, healthcare-associated *C. difficile* infection; NAAT, nucleic acid amplification test. †Sum of HA-CDI and CA-CDI cases might not equal number of overall CDI cases because of missing information in the registry. ‡Antimicrobial drug use 8 weeks before diagnosis was stratified into high risk (floroquinolones, cephalosporins, and clindamycin); medium risk (penicillins, macrolides, and sulfonamides); and low risk (tetracyclines).  §Defined as reappearance of symptoms after initial resolution and a positive CDI test result.

### Clinical Characteristics and Outcomes of HA-CDI and CA-CDI

We studied demographic and clinical characteristics in CDI patients of the 2 epidemiologic categories. Charlson comorbidity scores and proportions with severe CDI were similar between the 2 groups. Median ages (IQR) were 79 (65–86) years for patients with HA-CDI and 75 (57–85) years for patients with CA-CDI. For the CA-CDI group, male sex (41.2%), residence in a home for the elderly (24.9%), and exposure to high-risk antimicrobial drugs (36.6%) were less common than for HA-CDI group. The 30-day all-cause mortality rates were 9.8% for the CA-CDI group and 23.2% for the HA-CDI group, and the 60-day recurrence rates were 3.0% for the CA-CDI group and 8.0% for the HA-CDI group. However, a higher proportion of patients with CA-CDI than patients with HA-CDI had inflammatory bowel diseases (4.4% vs. 0.4%) (Table 2).

### Risk Factors for Death and Recurrence

Logistic regression modeling suggested that advanced age (adjusted odds ratio [OR] 5.04, 95% CI 3.88–6.55), non-metastatic tumor (adjusted OR 1.60, 95% CI 1.45–1.75), and healthcare-associated infection (adjusted OR 1.55, 95% CI 1.12–1.44) were the major predictors for death in 30 days. Other risk factors, including residence in a home for the elderly, exposure to high-risk antimicrobial drugs, and having renal diseases, each increased the risk for CDI by 39% to 47% (Table 3).

**Table 3:** Association between 30-day all-cause deaths and potential independent variables for patients with *Clostridium difficile* infections, Hong Kong, China, 2006–2014*

Variable	Univariate analysis			Multivariate analysis†		
	β	OR (95% CI)	p value	β	Adjusted OR (95% CI)	p value
Age, y						
<44	NA	1.0	NA	NA	1.0	NA
45−64	0.38	1.46 (1.34–1.58)	<0.01	0.39	1.48 (1.35–1.62)	<0.01
65−84	1.02	2.77 (2.45–3.14)	<0.01	0.99	2.69 (2.34–3.08)	<0.01
>85	1.77	5.87 (4.57–7.56)	<0.01	1.62	5.04 (3.88–6.55)	<0.01
Male sex	0.10	1.11 (1.03–1.19)	0.01	0.17	1.18 (1.09–1.28)	<0.01
Resident of home for elderly persons	0.55	1.73 (1.59–1.87)	<0.01	0.33	1.39 (1.27–1.52)	<0.01
Severe disease‡	−0.01	0.99 (0.92–1.08)	0.94	NA	NA	NA
Antimicrobial drug use§						
High-risk drug	0.62	1.85 (1.68–2.04)	<0.01	0.34	1.40 (1.26–1.56)	<0.01
Medium-risk drug	0.17	1.18 (1.09–1.28)	<0.01	0.10	1.11 (1.02–1.21)	0.02
Low-risk drug	0.24	1.27 (0.94–1.72)	0.12	NA	NA	NA
Diagnostic test						
Bacterial culture	NA	1.0	NA	NA	1.0	NA
Toxin detection	−0.33	0.72 (0.65–0.79)	<0.01	−0.25	0.78 (0.71–0.86)	<0.01
NAAT	−0.13	0.88 (0.80–0.97)	<0.01	-0.05	0.95 (0.86–1.05)	0.34
Use of proton-pump inhibitor	0.37	1.44 (1.34–1.56)	<0.01	0.24	1.27 (1.17–1.38)	<0.01
Use of histamine-2 receptor antagonist	0.15	1.16 (1.08–1.25)	<0.01	0.11	1.12 (1.03–1.21)	<0.01
Healthcare-associated disease	0.63	1.88 (1.65–2.13)	<0.01	0.44	1.55 (1.12–1.44)	<0.01
Concurrent condition						
Myocardial infarction	0.44	1.54 (1.37–1.74)	<0.01	0.24	1.27 (1.12–1.44)	<0.01
Cerebrovascular disease	0.10	1.11 (1.02–1.20)	0.01	0.14	1.14 (1.05–1.25)	<0.01
Chronic lung disease	0.08	1.08 (0.97–1.19)	0.16	NA	NA	NA
Diabetes mellitus	0.06	1.06 (0.96–1.17)	0.24	NA	NA	NA
Renal disease	0.31	1.36 (1.25–1.48)	<0.01	0.39	1.47 (1.34–1.61)	<0.01
Nonmetastatic tumor	0.18	1.2 (1.10–1.31)	<0.01	0.47	1.60 (1.45–1.75)	<0.01
AIDS	0.11	0.59 (0.31–1.12)	0.11	NA	NA	NA
Inflammatory bowled disease	−1.39	0.25 (0.13–0.49)	<0.01	−0.36	0.70 (0.35–1.41)	0.32

*NA, not applicable; NAAT, nucleic acid amplification test. †p = 0.74 by Hosmer-Lemeshow test. ‡Severe disease was diagnosed according to the according to criteria of Cohen et al (*13*).  §Antimicrobial drug use 8 weeks before diagnosis was stratified into high risk (floroquinolones, cephalosporins, and clindamycin); medium risk (penicillins, macrolides, and sulfonamides); and low risk (tetracyclines).

When we considered the 60-day disease recurrence, Cox regression analysis showed that the use of a toxin detection assay (adjusted hazard ratio 1.79, 95% CI 1.53–2.11) and healthcare-associated infection (adjusted hazard ratio 1.52, 95% CI 1.06–2.20) were the major predictors. Other risk factors included severe CDI and exposure to high-risk antimicrobial drugs (Table 4). When compared with incident cases, we found that the odds for recurrence among the first recurrent cases was 1.57 (95% CI 1.35–1.82). These odds increased to 2.10 (95% CI 1.62–2.74) after 2 recurrent episodes, and further increased by ≈10% to 2.22 (95% CI 1.38–3.57) after the third episode.

**Table 4:** Cox proportional hazard regression analysis of potential independent variables associated with time to recurrence of *Clostridium difficile* infections, Hong Kong, China, 2006–2014*

Variable	Univariate analysis			Multivariate analysis		
	β	Hazard ratio (95% CI)	p value	β	Adjusted hazard ratio (95% CI)	p value
Age, y						
<44	NA	1.0	NA	NA	1.0	NA
45−64	0.14	1.15 (0.86–1.55)	0.34	0.01	1.00 (0.74–1.36)	0.99
65−84	0.29	1.33 (0.01–1.75)	0.04	0.04	1.04 (0.78–1.38)	0.81
>85	0.41	1.50 (1.14–1.98)	<0.01	0.16	1.17 (0.87–1.56)	0.29
Male sex	−0.02	0.99 (0.88–1.10)	0.79	NA	NA	NA
Resident of home for elderly persons	−0.02	0.98 (0.87–1.11)	0.99	NA	NA	NA
Severe disease†	0.32	1.38 (1.22–1.55)	<0.01	0.35	1.41 (1.26–1.59)	<0.01
Antimicrobial drug use‡						
High-risk drug	0.49	1.55 (1.36–1.77)	<0.01	0.32	1.37 (1.20–1.57)	<0.01
Medium-risk drug	0.41	1.51 (1.33–1.72)	0.01	−0.01	0.99 (0.86–1.16)	0.96
Low-risk drug	0.11	1.12 (0.69–1.83)	0.66	NA	NA	NA
Diagnostic test						
Bacterial culture	NA	1.0	NA	NA	1.0	NA
Toxin detection	0.69	1.89 (1.62–2.21)	0.01	0.57	1.79 (1.53–2.11)	<0.01
NAAT	0.27	1.31 (1.12–1.52)	0.01	0.23	1.26 (1.08–1.47)	<0.01
Use of proton-pump inhibitor	0.08	1.09 (0.97–1.22)	0.16	NA	NA	NA
Use of histamine-2 receptor antagonist	0.18	1.19 (1.07–1.34)	0.01	0.09	1.09 (0.97–1.22)	0.15
Healthcare-associated disease	0.49	1.65 (1.15–2.35)	0.01	0.42	1.52 (1.06–2.20)	0.02
Concurrent condition						
Myocardial infarction	0.09	1.10 (0.92–1.32)	0.32	NA	NA	NA
Cerebrovascular disease	0.49	1.63 (1.46–1.82)	<0.01	−0.15	0.86 (0.74–0.99)	0.04
Chronic lung disease	−0.05	0.95 (0.82–1.12)	0.56	NA	NA	NA
Diabetes mellitus	0.08	1.08 (0.94–1.24)	0.30	NA	NA	NA
Renal disease	0.05	1.05 (0.92–1.20)	0.47	NA	NA	NA
Nonmetastatic tumor	−0.24	0.79 (0.69–0.90)	0.01	−0.15	0.86 (0.74–0.99)	0.04
AIDS	−0.58	0.56 (0.18–1.74)	0.32	NA	NA	NA
Inflammatory bowel disease	0.14	1.15 (0.62–2.14)	0.66	NA	NA	NA

*NA, not applicable; NAAT, nucleic acid amplification test. †Severe disease was diagnosed according to the according to criteria of Cohen et al (*13*).  ‡Antimicrobial drug use 8 weeks before diagnosis was stratified into high risk (floroquinolones, cephalosporins, and clindamycin); medium risk (penicillins, macrolides, and sulfonamides); and low risk (tetracyclines).

### Secular Changes in Mortality and Recurrence Rates

**Figure 2:**
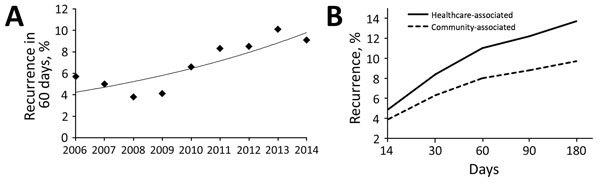
*Clostridium difficile* infections in adults, Hong Kong, China, 2006–2014. A) Prevalence of 60-day recurrence increased significantly (p<0.001 by χ^2^ test for trend. B) Recurrence rates, which were higher for healthcare-associated infections.

During 2006−2014, the crude 30-day all-cause mortality rate decreased slightly from 25.7% to 21.0% (OR 0.77, 95% CI 0.64–0.92; p = 0.01). Healthcare-associated case-patients in the 2014 cohort had an ≈20% reduced risk for death (OR 0.80, 95% CI 0.67–0.97; p = 0.02). The reduction was more apparent among the community-associated case-patients (OR 0.30, 95% CI 0.17–0.51; p<0.01). Despite the decreasing mortality rate, the recurrence rate increased significantly from 5.7% in 2006 to 9.1% in 2014 (p<0.01 by χ^2 ^test for trend) (Figure 2, panel A). This increase represented an ≈70% increase in the recurrence rate (OR 1.66, 95% CI 1.23–2.24). Further analysis suggested that the prevalence of severe disease, change of diagnostic test used, and exposure to proton-pump inhibitor changed over time (Table 5). We observed the same increasing trend for CA-CDI (2.5% vs. 5.7%) and HA-CDI (5.7% vs. 9.5%) during 2006−2014. Most recurrences occurred within 60 days after completion of initial treatment (Figure 2, panel B).

**Table 5:** Temporal change in exposure prevalence for patients with *Clostridium difficile* infections in association with trend in recurrence, Hong Kong, China, 2006–2014*

Exposure	Period, prevalence (standardized Pearson residuals), %			Absolute residual difference, %†	p value‡
	2006–2008	2009–2011	2012–2014		
Age, y					
<44	18.3 (0.4)	34.9 (3.0)	46.8 (−2.5)	2.9	<0.01
45–64	17.4 (−0.6)	32.5 (2.6)	50.1 (−1.7)	1.1	<0.01
65–84	19.2 (2.7)	28.9 (−1.4)	51.9 (−0.5)	3.2	<0.01
>85	16.1 (−3.0)	28.4 (−1.8)	55.5 (3.1)	6.1	<0.01
Male sex	48.6 (0.1)	48.5 (0.0)	48.4 (−0.1)	0.2	0.97
Resident of home for elderly persons	27.6 (−2.6)	29.5 (−1.0)	31.6 (2.3)	4.9	<0.01
Severe disease	16.9 (10.5)	32.6 (2.6)	50.5 (−6.7)	17.2	<0.01
Antimicrobial drug use§					
High-risk drug	18.5 (1.5)	28.4 (−2.7)	53.1 (1.2)	0.3	0.65
Medium-risk drug	17.4 (−1.1)	28.1 (−3.6)	54.5 (3.3)	4.4	<0.01
Low-risk drug	12.6 (−1.9)	19.3 (−2.9)	152 (3.3)	5.2	<0.01
Diagnostic test					
Bacterial culture	18.9 (1.7)	29.0 (−1.0)	52.0 (−0.3)	2	<0.01
Toxin detection	36.0 (31.1)	35.0 (6.9)	28.9 (−23.4)	54.5	<0.01
NAAT	0 (−31.7)	25.7 (−5.7)	74.3 (22.9)	54.6	<0.01
Use of proton-pump inhibitor	35.8 (−9.0)	40.7 (−6.6)	55.2 (10.3)	19.3	<0.01
Use of histamine-2 receptor antagonist	54.7 (6.0)	46.1 (−0.9)	44.9 (−2.9)	8.9	<0.01
Healthcare-associated disease	18.1 (0.7)	30.0 (0.5)	51.9 (−0.7)	1.4	<0.01
Concurrent condition					
Myocardial infarction	5.3 (−7.2)	7.9 (−3.4)	11.7 (6.8)	14	<0.001
Cerebrovascular disease accident	28.8 (−3.4)	32.8 (0.5)	33.5 (1.7)	5.1	<0.01
Chronic lung disease	12.4 (−3.6)	14.5 (−1.0)	16.3 (2.9)	6.5	<0.01
Diabetes mellitus	20.5 (2.6)	19.0 (1.0)	17.3 (−2.3)	4.9	<0.01
Renal disease	18.4 (−4.8)	22.5 (−0.2)	24.3 (3.1)	7.9	<0.01
Nonmetastatic tumor	21.7 (−3.7)	27.1 (2.6)	25.2 (0.2)	3.9	0.01
AIDS	0.5 (0.2)	0.6 (0.8)	0.4 (−0.7)	0.9	0.55
Inflammatory bowel disease	0.5 (−2.0)	0.7 (−1.2)	1.1 (2.2)	4.4	0.01

*NAAT, nucleic acid amplification test. †Absolute difference between standardized Pearson residuals in 2012–2014 and 2006–2008. ‡By χ^2^ test for trend. §Antimicrobial drug use 8 weeks before diagnosis was stratified into high risk (floroquinolones, cephalosporins, and clindamycin); medium risk (penicillins, macrolides, and sulfonamides); and low risk (tetracyclines).

## Discussion

Our main finding was the increasing incidence of CDI during 2006−2014 by an adjustment rate of 26% annually for HA-CDI and CA-CDI in Hong Kong. This increase was less than the 39% annual increase in South Korea (*5*) but higher than the 24% annual increase in Australia (*7*). This difference might be partially caused by increasing clinical suspicion, introduction of sensitive diagnostic tests in 2010, an aging population, or endemicity and virulence of the *C. difficile* bacterium. Prescriptions for antimicrobial drugs in Hong Kong have been closely monitored, and relevant use guidelines are available (*16*). In the United Kingdom and Finland, introduction of antimicrobial drug stewardship programs has considerably reduced CDI in hospitals (*2*,*4*).

In our study, the aging population contributed to the temporal increase. Advanced age is a well-established risk factor for CDI; observed a 2% increased risk for disease for each additional year of age (*17*). In our population, the incidence rate for elderly persons >75 years of age was higher than that for other age groups. In Hong Kong, the population is aging rapidly because of increased life expectancy and reduced birthrate. According to the latest United Nations report, the life expectancy at birth in Hong Kong is the longest in the world (*18*). Given the predicted increase in elderly persons >75 years of age from 7.3% in 2014 to 17.8% in 2041, the incidence of CDI is expected to reach 75.86 cases/100,000 persons (*19*). In addition, our logistic regression analysis showed that mortality and recurrence rates were much higher in elderly patients. Thus, the incidence of CDI might be expected to further increase, which represents a large burden on the healthcare system. Relevant surveillance should be enhanced and public health measures should be incorporated to reduce the disease burden, especially in places with an aging population.

Stratification of cases into epidemiologic categories showed that healthcare-associated infections contributed >90% of all incident cases detected. This contribution was considerably higher than those in some studies reporting up to 70% cases of healthcare origin (*1*,*2*,*7*). However, our rate was comparable to rates reported in 2 other studies in Asia (*10*,*20*). These findings highlight possible discrepant epidemiologic etiologies of the infection at different locations (*21*). Recent emergence of *C. difficile* ribotype 002 in the region may also account for the discrepancy (*15*,*22**,**23*). This common circulating ribotype has a greater propensity to sporulate and produce toxins. These spores and toxin producers might facilitate bacterial dissemination in the hospital environment and increase the number of symptomatic patients. Because data were available for tertiary care settings in our study, community cases might not be recognized unless patients became sufficiently ill for hospital admission. A delay in diagnosis is rather common for CA-CDI (*10*).

Emergence of CA-CDI has been increasingly recognized. Persons with this type of infection have had no traditional risk factors associated with nosocomial infection. The younger age of the disease population has led to concern over loss of productivity and years of life lost (*3*,*24*). Despite the relatively low prevalence, we observed a higher increase of incidence for the community than that for healthcare settings. Risk factors for acquisition of CDI in the community are unclear, but bacterial, host, and environmental factors have been suggested to play a role (*25*). Contamination of retail meats with *C. difficile* spores might represent a potential reservoir for infection of humans (*26**,**27*). Contact with contaminated raw meat has been recognized as a risk factor for nasal colonization with *Staphylococcus aureus* in humans (*28*). Molecular typing of *C. difficile* isolates from contaminated raw meat and those from exposed personnel might identify a novel reservoir for asymptomatic carriage. Although not yet proven for the community, asymptomatic carriers have been shown to contribute to transmission of *C. difficile* strains in healthcare facilities (*29*).

The 30-day all-cause mortality rate decreased slightly during the study period. This finding was consistent with other reports in which 20%−30% reductions were detected (*5*,*30*). When compared with mortality rates for CA-CDI, mortality rates at different times were consistently higher in the HA-CDI group. This finding is consistent with another report in which a 4-fold higher case-fatality rate for HA-CDI was observed (*2*). Consistently, logistic regression analysis suggested that healthcare-associated cases, in addition to advanced age, were the major risk factors for 30-day all-cause mortality rates.

The recurrence rate for our population was considerably lower than rates reported for Western countries (*3*,*5*,*8*). Although this discrepancy would require further validation, our data might indicate lower disease recurrence rates for Asia. Because various therapies other than antimicrobial drugs, including colectomy and fecal microbiota transplant, have been considered for managing recurrent CDI cases, this lower recurrence rate might indicate a need to reconsider the risk−benefit balance when deciding which therapies to use. Further studies are warranted to investigate differences in recurrence rates.

We also observed a major increase in recurrence rate over the study period. The factors accounting for this change were multifactorial and might include change of diagnostic method used over time, increasing prevalence of severe disease, and exposure to proton-pump inhibitors. A systematic review of 68 studies concluded that use of antimicrobial drugs after diagnosis, in addition to older age, were major risk factors for recurrence (*31*). However, in our study, we did not investigate exposure to antimicrobial drugs after diagnosis. Similarly, increased ward-level prescriptions for antimicrobial drugs have been shown to increase CDI in hospitalized patients (*32*).

Our study was strengthened by use of a large territory-wide population. Because public hospitals provide >90% of the entire inpatient service in Hong Kong, our study was representative of the region. Our study also investigated the temporal trend of burden of CDI. All data, including demographics, laboratory findings, and clinical records, were objectively recorded in a database. Thus, there was no cause for concern regarding recall bias.

Nevertheless, our study had some limitations. First, the study relied on inpatient data for which cases diagnosed in outpatient clinics might have been missed. This limitation might lead to underestimation of disease burden, particularly for community-associated case-patients with milder disease, who have been managed as outpatients without the need for hospitalization. This limitation might have also skewed clinical characteristics and outcomes for this group, although all patients with severe cases requiring hospitalization would have been represented in our data.

Second, because of lack of coding, our database was not able to capture fecal microbiota transplants as a novel therapy for recurrent CDI. Nevertheless, because of operational and logistic difficulties, fecal microbiota transplant has been used sparsely for selected patients (*33*). This procedure was unlikely to have caused any major changes in overall disease epidemiology. 

Third, we did not include repeated exposure to antimicrobial drugs after initial CDI diagnosis in our database. This limitation might potentially serve as a major factor for subsequent disease recurrence.

Fourth, as with other retrospective studies (*1*,*2*,*7*,*34**,**35*), the diagnosis of CDI in our study was based on different laboratory methods, including bacteriological culture, toxin detection, and molecular assays. Given the variable sensitivity and specificity of these tests, this limitation could have biased estimation of disease incidence. Cases of pseudomembranous colitis diagnosed only by histologic analysis might have been missed. Thus, the incidence of CDI might be underestimated.

In conclusion, the incidence of CDI is increasing at a rapid rate in Hong Kong. Further surveillance of this infection in this area is urgently needed.
